# Dendritic Cell‐Mimicking Nanoparticles Promote mRNA Delivery to Lymphoid Organs

**DOI:** 10.1002/advs.202302423

**Published:** 2023-10-22

**Authors:** Yiming Cao, Jinrong Long, Huisheng Sun, Yiqi Miao, Ye Sang, Haitao Lu, Changxiao Yu, Zhen Zhang, Lin Wang, Jing Yang, Shengqi Wang

**Affiliations:** ^1^ Bioinformatics center of AMMS Beijing 100850 P. R. China; ^2^ Beijing Institute of Radiation Medicine Beijing 100850 P. R. China

**Keywords:** delivery, dendritic cell membrane, lymphoid organs, mRNA vaccine

## Abstract

Spleen and lymphoid organs are important targets for messenger RNA (mRNA) delivery in various applications. Current nanoparticle delivery methods rely on drainage to lymph nodes from intramuscular or subcutaneous injections. In difficult‐to‐transfect antigen‐presenting cells (APCs), such as dendritic cells (DCs), effective mRNA transfection remains a significant challenge. In this study, a lymphatic targeting carrier using DC membranes is developed, that efficiently migrated to lymphoid organs, such as the spleen and lymph nodes. The nanoparticles contained an ionizable lipid (YK009), which ensured a high encapsulation efficacy of mRNA and assisted mRNA with endosomal escape after cellular uptake. Dendritic cell‐mimicking nanoparticles (DCMNPs) showed efficient protein expression in both the spleen and lymph nodes after intramuscular injections. Moreover, in immunized mice, DCMNP vaccination elicited Spike‐specific IgG antibodies, neutralizing antibodies, and Th1‐biased SARS‐CoV‐2‐specific cellular immunity. This work presents a powerful vaccine formula using DCMNPs, which represents a promising vaccine candidate for further research and development.

## Graphical abstract

Dendritic cell‐mimicking nanoparticles (DCMNPs), which can effectively deliver mRNA to lymphatic organs, are developed as a novel mRNA delivery carrier. The DCMNPs are formed by encapsulating mRNA in a carrier composed of DC membrane, ionizable lipid YK009 and DOPE. DCMNPs with SARS‐CoV‐2 Spike protein mRNA can triggered a robust neutralizing antibody and SARS‐CoV‐2‐specific cell immunity in mice.

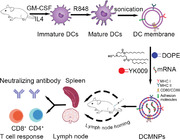

## 1 Introduction

Secondary lymphoid organs, including lymph nodes, spleen, adenoids, and other mucosal‐associated lymphoid tissue, provide an environment that enables lymphocytes to interact with each other, accessory cells, and antigens, resulting in the initiation of an antigen‐specific primary immune response.^[^ ^1^ ^]^ As such, efficient delivery of mRNA to secondary lymphoid organs is highly desirable.^[^ ^2^ ^]^ Lipid nanoparticles (LNPs) have successfully achieved the clinical applications of mRNA therapeutics, showing notable success in COVID‐19 vaccines.^[^ ^3^ ^]^ However, most of the reported LNP formulations tend to accumulate in the liver upon systemic administration.^[^ ^4^ ^]^ Indeed, the first ionizable lipid FDA‐approved LNP drug, which was based on DLin‐MC3‐DMA as a key ingredient, was shown to be predominantly expressed in the liver.^[^ ^5^ ^]^ Previous reports have documented the effectiveness of MC3‐based LNPs for delivering mRNA.^[^ ^6^ ^]^ Clinically used LNPs using other ionizable lipids have also been shown to exhibit strong mRNA protein expression, including ALC‐0315 and SM‐102.^[^ ^7^ ^]^ Although these formulations have feasibility for mRNA therapy, their cytotoxicity, poor biological distribution, and lack of specific targeting remain challenges for LNPs.^[^ ^8^ ^]^ These problems may be due to LNPs requiring surface PEGylation to improve their cycle half‐life and reduce non‐specific clearance.^[^ ^9^ ^]^ Additionally, non‐immune cells at the injection site, including muscle cells and epithelial cells, take up the majority of the injected LNPs but do not participate in the antigen presentation that occurs in the draining lymph nodes.^[^ ^2^ ^]^ Moreover, due to reported adverse events caused by LNPs, such as inflammation and pain, the safety of LNPs is also of concern.^[^ ^10^ ^]^ Therefore, it is necessary to develop effective and safe vaccines that allow the targeted delivery of mRNA vaccines to tissues with abundant immune cells. Several studies have attempted to achieve non‐liver delivery of mRNA, such as delivery to the spleen, lung, or lymphatics, either actively or passively by manipulating the properties of LNPs.^[^ ^11^ ^]^ However, there remains a need for more effective delivery strategies to target immune cells.

In recent years, biomimetic designs based on cell membrane coatings have gradually emerged. As a natural analog of liposomes, a phospholipid bilayer structure can endow nanocarriers with the inherent functionality of the cell membrane. Bioinspired nanoparticles engineered with cell membranes show cell‐like functionality and represent a novel strategy for drug delivery.^[^ ^12^ ^]^ Cell membrane‐coated nanoparticles can integrate the advantages of various proteins and molecules into the cell membranes, conferring them with better biocompatibility and lower immunogenicity, and enabling them to escape clearance by the immune system.^[^ ^13^ ^]^ Indeed, erythrocyte membranes have been used to prolong nanoparticle circulation time,^[^ ^14^ ^]^ whereas cancer cell membranes and platelet membranes have been leveraged for targeted drug delivery.^[^ ^15^ ^]^ Dendritic cells (DCs), known as antigen‐presenting cells, activate CD8^+^ or CD4^+^ T cells by cross‐presenting antigens on major histocompatibility complex class I (MCH‐I) or II (MHC‐II) molecules, respectively. DCs, which possess co‐stimulatory molecules and effectively migrate through lymphoid tissue to encounter T cells, are considered the best activators and inducers of naive T and B cell proliferation. DCs can internalize and destroy invasive microorganisms through phagocytosis, and effectively internalize pathogens and present the pathogen peptides on their cell surface to activate adaptive immune system cells. After engulfing the pathogen, DCs migrate to a lymphoid site within the blood or lymph.^[^ ^1a^ ^]^ Given the central role of DCs in controlling the immune response, DC membrane coated‐nanoparticles have been studied in the context of cancer immunotherapy, where they have been shown to strongly inhibit tumor growth and induce memory T cell activation for long‐term protective immunity.^[^ ^16^ ^]^ These studies have demonstrated that the application of DC membranes as a vaccine platform in immunotherapy can stimulate humoral immunity. DC membranes may also have the potential to stimulate cellular immunity for mRNA therapy, although there has been limited progress in this area at this time.

**Figure 1:**
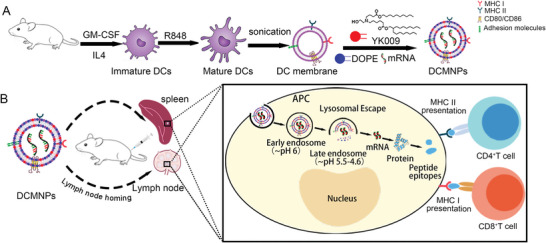
DC‐mimicking nanoparticles (DCMNPs) promote mRNA delivery to lymphoid organs. A) Schematic illustration of the preparation of DCMNPs. B) DCMNPs feature a nanoscale size and lymph node homing characteristics, promoting mRNA accumulation in the lymph nodes and spleen. Following capture of the nanoparticles by antigen‐presenting cells, particularly DCs, the ionizable lipid (YK009) further improves mRNA endosomal escape properties and enhances cytosolic delivery.

In this work, by leveraging the lymph node homing characteristics of DCs, we established a flexible mRNA vaccine platform based on the DC membrane. mRNA was encapsulated in a carrier composed of a DC membrane, ionizable lipid (YK009; Figure S1, Supporting Information), and 1,2‐dioleoyl‐sn‐glycero‐3‐phosphoethanolamine (DOPE). The DC‐mimicking nanoparticles (DCMNPs) showed advantages in mRNA delivery from several aspects. First, the ionizable lipids ensured a high loading efficiency of mRNA, with the positively charged YK009 capable of interacting with the negatively charged mRNA. Second, the DC membrane conferred lymph node homing characteristics to the nanoparticles, improved the distribution of mRNA in vivo, and promoted mRNA accumulation in the lymph nodes and spleen. Moreover, when the nanoparticles were captured by APCs, particularly DCs, the ionizable lipids further improved the endosomal escape properties and cytosolic delivery of mRNA. Additionally, mRNA based on the SARS‐CoV‐2 Spike protein mRNA‐4138 was designed and synthesized to test the effectiveness of the DCMNPs as an mRNA vaccine carrier. Our results suggest that the DCMNP vaccine had a good biodistribution and high delivery efficiency. Moreover, our results confirmed that the DCMNP (mRNA‐4138) vaccine triggered a robust neutralizing antibody and SARS‐CoV‐2‐specific cell immunity. **Figure** **1**

## 2 Results

### 2.1 Characterization of the DC Membrane (DCM) and Optimization of DCMNPs

**Figure 2:**
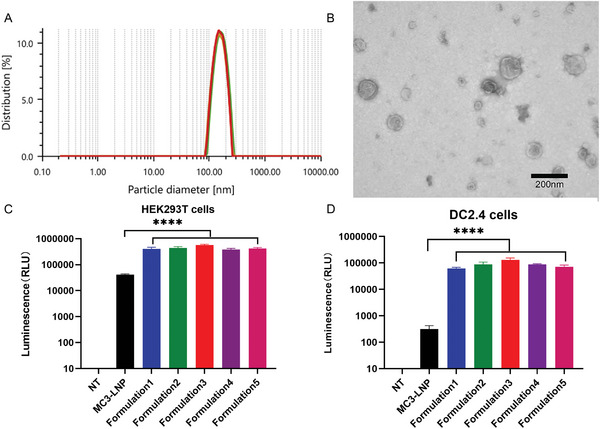
Characterization of the DC membrane (DCM) and optimization of DCMNPs. A) The hydrodynamic diameter of the DCM was 168.48 ± 5.20 nm. B) Transmission electron microscopy image of DCM. Scale bar: 200 nm. C) Optimization of DCMNPs (Fluc mRNA) in HEK293T cells. D) Optimization of DCMNPs in DC2.4 cells. Luciferase expression of different DCMNP formulations after 24 h in vitro. Formulation 1: DCM/mRNA = 1:1 (volume/mass), Formulation 2: DCM/mRNA = 2:1, Formulation 3: DCM/mRNA = 3:1, Formulation 4: DCM/mRNA = 4:1, Formulation 5: DCM/mRNA = 5:1. Data are shown as the mean ± SEM (*n* = 3) and analyzed by one‐way ANOVA with multiple comparisons tests (****p < 0.0001).

Bone marrow‐derived dendritic cells (BMDCs) were extracted from mice and stimulated with resiquimod (R848) to induce maturation. The expressions of CD80 and CD86 were detected by flow cytometry. Flow cytometry analysis results showed that the expression of costimulatory molecules was significantly upregulated in DCs stimulated by R848 (Figure S3, Supporting Information). Subsequently, the DCM was isolated and purified. The hydrodynamic diameter of the DCM was 168.48 ± 5.20 nm (**Figure** **2**A). The DCM morphology was examined by negative stain transmission electron microscopy (TEM), which revealed that most of the DCM had a spherical shape with a particle size close to the measured value (Figure 2B). To obtain the optimal system formulation, DCMNPs using firefly luciferase mRNA (Fluc mRNA) as a reporter were transfected into HEK293T (Figure 2C) and DC2.4 cells (murine bone marrow‐derived DC line), with MC3‐LNP set as the reference (Figure 2D). As a result, DCMNPs demonstrated a luciferase expression level that was ≈14 times higher than that of MC3‐LNP in HEK293T cells. Similarly, DCMNPs demonstrated an expression level that was ≈400 times higher than that of MC3‐LNP in DC2.4 cells and had a higher transfection efficiency.

### 2.2 Fabrication and Characterization of DCMNPs

**Figure 3:**
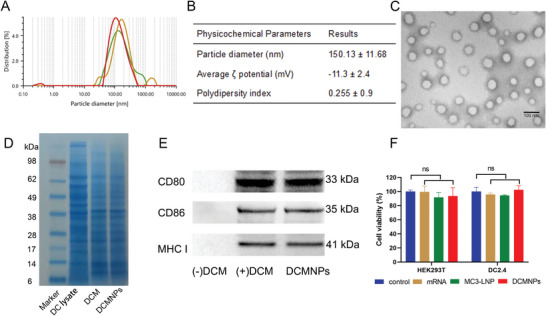
Characterization of DCMNPs. A) The hydrodynamic diameter of DCMNPs was 150.13 ± 11.68 nm. B) The physicochemical parameters of DCMNPs. C) Transmission electron microscopy image of DCMNPs. Scale bar: 100 nm. D) SDS‐PAGE analysis of the total protein. Proteins from the DC lysate, DCM, and DCMNPs. E) Western blot analysis of the expression of membrane‐specific proteins, including CD80, CD86, and MHC I. F) Cell viability of HEK293T and DC2.4 cells after transfection with DCMNPs for 24 h. Data are shown as the mean ± SEM (*n* = 3) and analyzed by one‐way ANOVA with multiple comparisons tests (ns: Not significant, *p* >0.05).

Using the optimal formulation (Formulation 3), which was determined based on the highest transfection efficiency (Figure 2C,D), the hydrodynamic diameter of the DCMNPs was 150.13 ± 11.68 nm, and the zeta potential was −11.3 ± 2.4 mV. DCMNPs were further characterized using TEM. The morphologic TEM image shown in **Figure** **3**C suggested that DCMNPs were able to form spherically shaped and homogenously distributed nanocomplexes. The encapsulation efficiency of the Fluc mRNA was determined to be 70.84%. The profiles of the membrane proteins on DCM and DCMNPs were analyzed by SDS‐PAGE. The preparations containing DCM showed protein bands at different molecular weight marker protein positions, and their protein profile closely matched that of the purified membranes (Figure 3D). Moreover, CD80, CD86, and MHC‐I were identified on the DCMNPs due to the fused DCM. The main functional membrane proteins on the surface of the DCMNPs were similar to those of the DCM (Figure 3E). Additionally, the cytotoxicity of DCMNPs was evaluated in HEK293T and DC2.4 cells, with the results indicating that DCMNPs exhibited low levels of cytotoxicity to HEK293T and DC2.4 cells (Figure 3F).

### 2.3 Protein Expression of DCMNPs In Vitro

**Figure 4:**
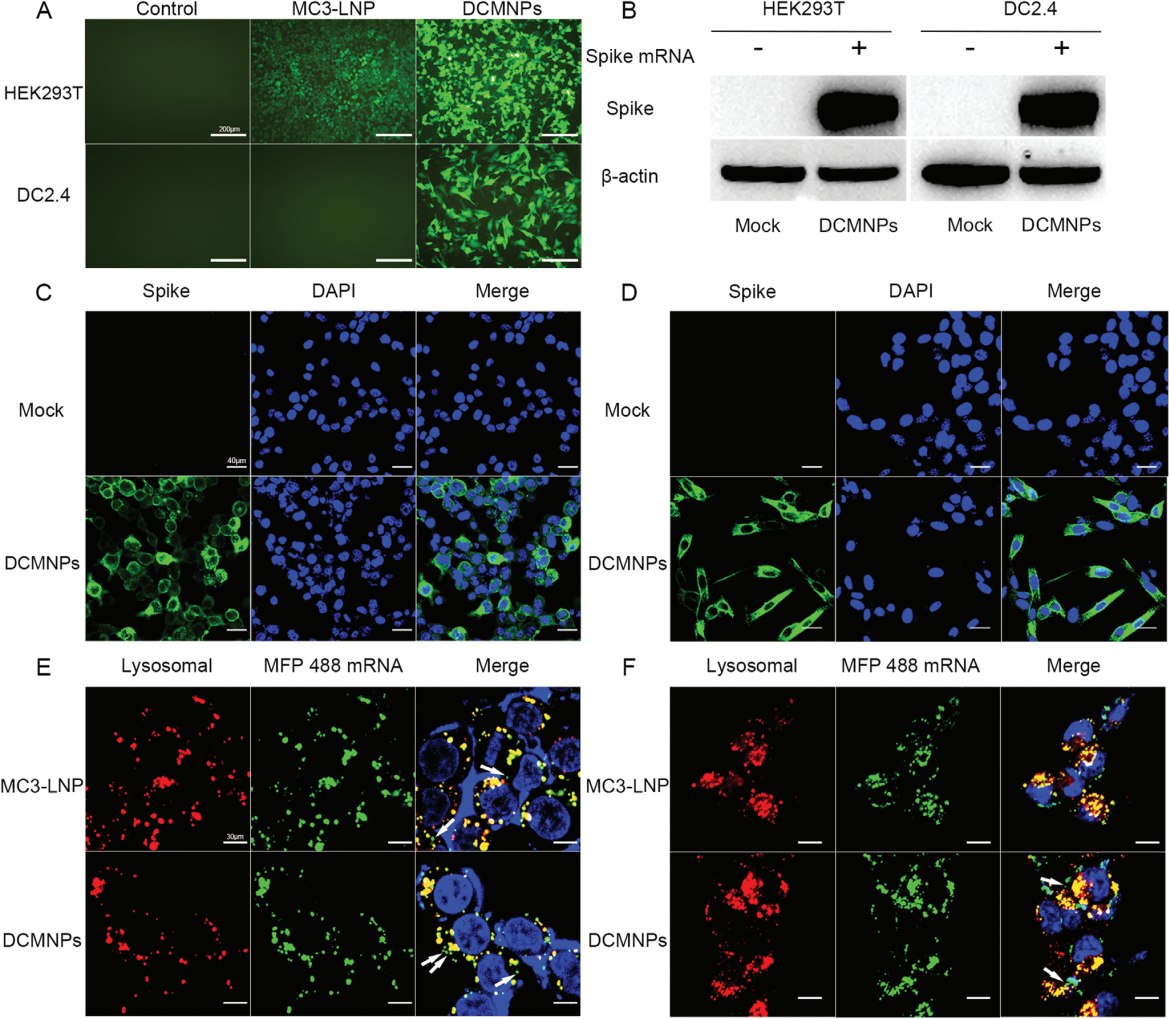
Protein expression of DCMNPs in vitro. A) eGFP mRNA was transfected into HEK293T and DC2.4 cells using DCMNPs or MC3‐LNP. Strong expression of eGFP in HEK293T cells and DC2.4 cells following incubation with DCMNPs for 24 h. Scale bar: 200 µm. B) Western blotting analysis of SARS‐CoV‐2 Spike protein expression in HEK293T and DC2.4 cells at 24 h post transfection. C and D) Indirect immunofluorescence analysis of SARS‐CoV‐2 Spike protein expression in HEK293T and DC2.4 cells at 24 h post transfection using DCMNPs. Scale bar: 40 µm. E) HEK293T and F) DC2.4 cell confocal images showing intracellular distribution of mRNA (green) and lysosomes (red) at 6 h post transfection. The white arrow indicates the lysosomal escape of the delivered mRNA. Scale bar: 30 µm.

Next, we quantitatively evaluated the ability of DCMNPs to successfully deliver functional mRNA cargo for protein translation. First, DCMNPs were formulated with enhanced green fluorescent protein (eGFP) mRNA. We detected strong expression of eGFP in HEK293T cells and DC2.4 cells following incubation with DCMNPs for 24 h. MC3‐LNP was only expressed in HEK293T cells and only minimally expressed in DC2.4 cells (**Figure** **4**A). Flow cytometry analysis results showed that DCMNPs induced higher eGFP expression and fluorescence intensity than that of the MC3‐LNP reference group in vitro, particularly in DC2.4 cells (Figure S4B and C, Supporting Information), which was similar to the results of the Fluc mRNA transfections (Figure S4D and E, Supporting Information). Using this system, a COVID‐19 mRNA vaccine was prepared using mRNA‐4138 encoding the full‐length Spike protein as the immunogen (Figure S2, Supporting Information), the expression of which was confirmed by western blot analysis (Figure 4B). Additionally, the mRNA‐4138 molecule was tested by indirect immunofluorescence, and stable and efficient Spike protein expression was confirmed in HEK293T cells (Figure 4C), and DC2.4 cells (Figure 4D) following transfection. The results showed that DCMNPs effectively encapsulated the COVID‐19 mRNA vaccine and transfected it into cells with subsequent expression of the Spike protein, suggesting that DCMNPs could be used for vaccine delivery. Endosomal escape of the nanocomplex was essential for efficient mRNA delivery to avoid intracellular degradation of mRNA in the endosome‐lysosome pathway. We investigated the lysosome escape behaviors of the MFP‐488 fluorescently labeled mRNA‐4138 and the lysosomes stained with Lysotracker red in DC2.4 cells using a high content imaging system (PerkinElmer Opera Phenix). The colocalization/lysosomal area was maximum at 1 h, and the colocalization/lysosomal area at 6 h are decreases than those at 1 h. The colocalization/lysosomal area gradually decreases with time suggesting that the DCMNPs could smoothly escape from the lysosomes (Figure S5, Supporting Information). The lysosomal escape of DCMNPs in HEK293T and DC2.4 cells was further tracked via fluorescent colocalization by confocal microscopy. The results showed that after 6 h of incubation, the signals of MFP488 (mRNA‐4138) and Lysotracker partially separated, demonstrating the successful lysosomal escape of DCMNPs (Figure 4E,F). The Pearson's correlation coefficients at 6 h were quantified as 0.38 in HEK293T and 0.42 in DC2.4 cells (Figure S6, Supporting Information). These results demonstrate that DCMNPs were efficiently taken up by cells and then underwent intracellular lysosomal escape, which is essential for successful mRNA translation.

### 2.4 Delivery and Biodistribution of DCMNPs In Vivo

**Figure 5:**
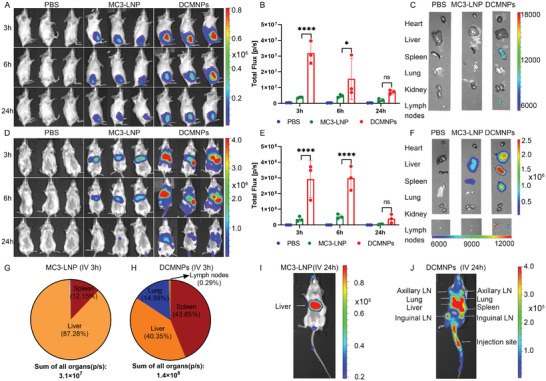
Delivery and biodistribution of DCMNPs in vivo. A) Whole‐body bioluminescent images were obtained and analyzed at 3, 6, and 24 h after intramuscular injection. B) Total luminescence flux of the whole‐body bioluminescence signal 3, 6, and 24 h after intramuscular injection. C) Ex vivo images of the major organs and lymph nodes after intramuscular injection for 3 h. D) Whole‐body bioluminescent images were obtained and analyzed at 3, 6, and 24 h after intravenous injection. E) Total luminescence flux of the whole‐body bioluminescence signal at 3, 6, and 24 h after intravenous injection. F) Ex vivo images of major organs and lymph nodes after intravenous injection for 3 h. G) Sum of all luminescence signals from all organs 3 h after intravenous injection of MC3‐LNP. H) Sum of all luminescence signals from all organs 3 h after intravenous injection of DCMNPs. I) Representative bioluminescent images 24 h after intravenous injection of MC3‐LNP. J) Representative bioluminescent images 24 h after intravenous injection of DCMNPs. These results demonstrated that DCMNPs effectively overcame tissue barriers to become enriched in peripheral lymph nodes. Data are shown as the mean ± SEM (*n* = 3) and were analyzed by two‐way ANOVA with multiple comparisons tests (ns: Not significant; *p* > 0.05; *p < 0.05; ****p < 0.0001).

Secondary lymphoid organs are important targets for mRNA delivery. Successful antigen presentation in vivo requires DCMNPs to target lymph nodes and make full contact with T cells. After confirming the successful protein translation in vitro, we next evaluated the ability of DCMNPs to achieve transfection and biodistribution in vivo. To evaluate the capability of DCMNP delivery in mice, Fluc mRNA was prepared with DCMNPs, and then DCMNPs were administered through different inoculation routes and biofluorescence imaging analysis was conducted. Biofluorescence whole‐body imaging was conducted at 3, 6, and 24 h after administration. Following intramuscular injection, luciferase was mainly expressed in the muscle at the injection site (**Figure** **5**A). To compare the expression level more accurately, we analyzed the whole‐body bioluminescence signal after intramuscular injection. As the results showed, the highest signals were detected at 3 h post injection, and the luminescence signal was nine‐fold stronger with DCMNPs than with MC3‐LNP (Figure 5B). Ex vivo imaging analysis of the DCMNPs showed that luciferase was also expressed in the spleen and inguinal lymph nodes near the injection site which was not detected with MC3‐LNP (Figure 5C).

We further evaluated the location of luciferase expression in mice after intravenous injection. As reported in the literature, MC3‐LNP showed maximum luciferase expression in the liver,^[^ ^2^, ^5^ ^]^ while DCMNPs showed luciferase expression in the spleen, liver, lung, and lymph nodes (Figure 5D). Quantification of the signals revealed that the total flux of the DCMNP group was more than eight‐fold higher at 3 h post injection than that of the MC3‐LNP group (Figure 5E). Next, we investigated the biodistribution of DCMNPs by imaging isolated major organs and lymph nodes at 3 h after intravenous injection. The results showed that luciferase was expressed in the spleen, liver, lung, and lymph nodes (Figure 5F). Additionally, the luminescence signals from all organs were measured at 3 h after intravenous injection and summed. As shown in Figure 5G,H, MC3‐LNP showed maximal luciferase expression in the liver (87.28%), whereas DCMNPs showed expression in the spleen (43.85%), liver (40.35%), lung (14.99%), and lymph nodes (0.29%). Furthermore, the expression of luciferase in lymph nodes, including axillary and inguinal lymph nodes, showed stronger fluorescence intensity at 24 h after intravenous injection, while no fluorescence signal was detected in MC3‐LNP (Figure 5I,J). Notably, DCMNPs promoted the targeting of mRNA to the spleen and lymph nodes far more efficiently than MC3‐LNP. To summarize, these results suggested that DCMNPs efficiently accumulated in the lymphoid organs and achieved targeted expression while maintaining a high transfection efficiency.

### 2.5 Efficacy of DCMNPs in Mice

**Figure 6:**
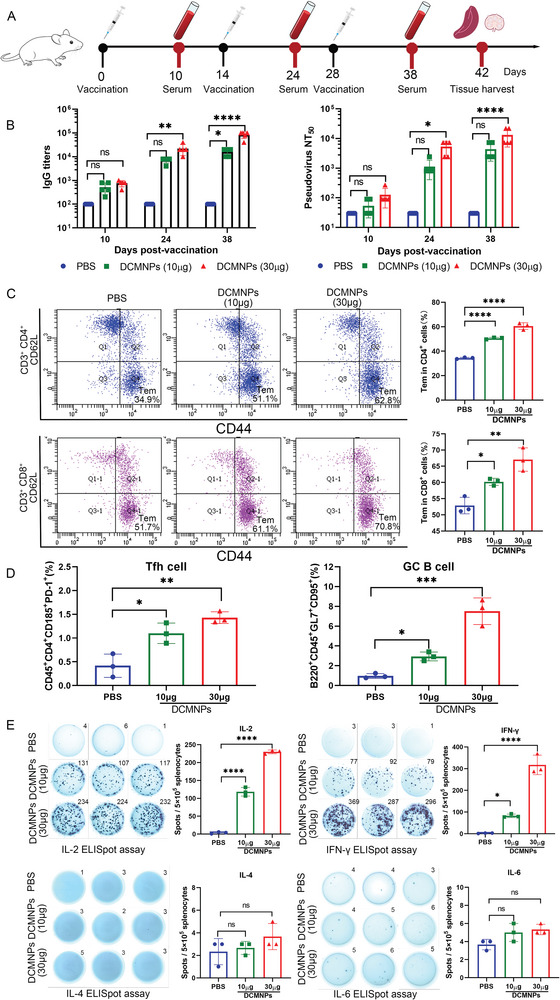
Efficacy of the DCMNP (mRNA‐4138) vaccine in mice. A) Schematic diagram of the immunization strategy, serum collection and immunogenicity detection scheme. B) Titers of Spike‐specific IgG antibodies determined by ELISA. The serum neutralization antibody titer (NT_50_) against the SARS‐CoV‐2 pseudovirus. Data are shown as the mean ± SEM (*n* = 5) and analyzed by two‐way ANOVA with multiple comparisons tests (ns: Not significant; *p* > 0.05; *p < 0.05; **p < 0.01; ****p < 0.0001). C) Flow cytometric detection of the SARS‐CoV‐2 Spike‐specific CD4^+^ and CD8^+^ effector memory T cells in splenocytes. D) Tfh and GC B cells in the lymph nodes of an immunized mouse were analyzed by flow cytometry. E) ELISPOT detection of IL‐2 (Th1), IFN‐γ (Th1), IL‐4 (Th2), and IL‐6 (Th2) released in splenocytes. Data are shown as the mean ± SEM (*n* = 3) and were analyzed by one‐way ANOVA with multiple comparisons tests (ns: Not significant; *p* > 0.05; *p < 0.05; **p < 0.01; ***p < 0.005; ****p < 0.0001).

To further verify whether DCMNPs could be used for delivery of mRNA vaccine and induce an immune response in vivo, BALB/c mice were immunized with three doses of 10 or 30 µg of mRNA‐4138 in a DCMNP vaccine through intramuscular administration, according to the immunization process illustrated in **Figure** **6**A. The titers of Spike‐specific IgG antibodies were determined by enzyme‐linked immunosorbent assay (ELISA) to evaluate the humoral immune response. After two booster immunizations with the same dose, the serum SARS‐CoV‐2 Spike‐specific IgG antibody levels in mice vaccinated with DCMNPs (mRNA‐4138) remarkably increased. After the third immunization, the endpoint titers in mice immunized with 10 or 30 µg of mRNA‐4138 in DCMNPs approached 1/16 000 and 1/84 000, respectively. The neutralizing antibodies with a 50% neutralization titer (NT_50_) approached 1/4374 and 1/13122, respectively (Figure 6B). Our results showed that immunization with three doses of the DCMNP (mRNA‐4138) vaccine induced high levels of SARS‐CoV‐2 Spike‐specific IgG antibodies and neutralizing antibodies.

Next, flow cytometry was performed to further investigate whether three doses of the DCMNP (mRNA‐4138) vaccine induced a specific T cell immune response in mice. The results showed a significant increase in SARS‐CoV‐2 Spike‐specific CD4^+^ and CD8^+^ effector memory T cells (Tem) (Figure 6C). We also evaluated the proportion of T follicular helper (Tfh) and germinal center (GC) B cells induced by the DCMNP (mRNA‐4138) vaccine. Compared to the phosphate buffered saline (PBS) group, the percentage of Tfh and GC B cells in the lymph nodes of DCMNP (mRNA‐4138) immunized mice was significantly increased (Figure 6D). Moreover, the results of enzyme‐linked immunospot (ELISPOT) assay revealed that the secretion of IL‐2 (Th1) and IFN‐γ (Th1) in splenocytes of the DCMNP (mRNA‐4138) vaccinated mice was significantly higher compared to those treated with PBS, but with no significant difference in IL‐4 (Th2) or IL‐6 (Th2) levels in the splenocytes of the DCMNP (mRNA‐4138) vaccinated mice (Figure 6E). Our results suggest that the DCMNP (mRNA‐4138) vaccine induced a strong Th1‐biased SARS‐CoV‐2‐specific cellular immune response.

### 2.6 Safety of DCMNPs in Mice

**Figure 7:**
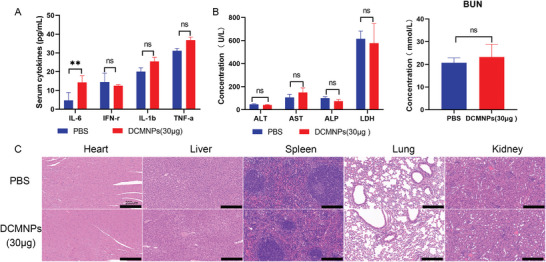
Safety of DCMNPs (30 µg mRNA‐4138) in mice. A) Cytokine stimulation by DCMNPs measured at 24 h. B) Blood biochemical indices measured at 24 h after DCMNP treatment. Data are shown as the mean ± SEM (*n* = 3) and were analyzed by two‐way ANOVA with multiple comparisons tests (ns: Not significant, *p* > 0.05; **p < 0.01). C) H&E staining of the major organ sections was performed 24 h after injection. Scale bar: 200 µm.

The ideal nanocarrier not only needs to have a high delivery efficiency, but also needs to have minimal toxicity and high biocompatibility. To evaluate the safety of DCMNPs in vivo, the level of several important cytokines, biochemical indices, and tissue histopathological damage were determined. Serum specimens were collected at 24 h post‐administration to detect the levels of several cytokines, including IL‐1b, TNF‐α IFN‐γ, and IL‐6. The results showed that the levels of IL‐1b, TNF‐α, and IFN‐γ did not significantly change in mice vaccinated with DCMNPs (mRNA‐4138) compared to the control. The level of IL‐6, a cytokine that is critical for early Tfh cell differentiation in mice, was slightly higher in the vaccinated mice (**Figure** **7**A).^[^ ^17^ ^]^ Moreover, it has been reported that the production of IL‐6 peaked at 4 h after immunization and remained elevated for at least 24 h, before beginning to decline after 48 h.^[^ ^18^ ^]^ The blood biochemical indices, including ALT, AST, ALP, LDH, and BUN were within the normal range (Figure 7B). Moreover, no obvious pathological damage was observed from hematoxylin and eosin (H&E) staining of the major organ sections (Figure 7C). These data indicated that DCMNPs can be used for safe and effective mRNA delivery in vivo.

## 3 Discussion

Many extracellular and intracellular barriers need to be overcome for mRNA formulations to function in vivo. First, mRNA needs to be protected from nuclease degradation in physiological fluids. Second, mRNA formulations need to reach the target tissue and then be internalized by the target cells. Finally, the mRNA molecule must escape from the endosome to the cytoplasm, where translation occurs.^[^ ^19^ ^]^ In recent years, cell membranes have emerged as potential vectors for nucleic acid‐based therapeutics due to their inherent biocompatibility, low immunogenicity, and their ability to cross physiological barriers.^[^ ^20^ ^]^ As the interface between cells and the external environment, cell membranes contain components that guide intercellular communication. Nanoparticles disguised by a cell membrane would be perceived as originating from the source cells, which provides superior drug delivery and has attracted much attention.^[^ ^21^ ^]^ Based on biocompatibility and bioavailability, the cell membrane has been considered a potential mRNA drug carrier. As DCs play a critical role in antigen presentation. mRNA complexes were decorated with ligands and scavenger receptors, such as mannose and langerin, to selectively target DCs.^[^ ^22^ ^]^ Ionizable lipids as a critical component of the formulation of LNPs, determine mRNA delivery, cellular uptake, endosomal escape, and reactogenicity.^[^ ^23^ ^]^ Due to the negative charge of cell membranes, we designed and screened an ionizable lipid and fused it with DC membranes to control and modify the performance of the cell membrane nanocarriers. We developed a DC‐mimicking nanoparticle delivery system for mRNA delivery. DC membranes endow the nanoparticles with lymph node homing characteristics, ensuring efficient targeting of nanoparticles to lymphoid organs. The ionizable lipids ensured high loading efficiency of mRNA and protected mRNA from degradation in vivo. The nanoparticles improved mRNA endosomal escape properties and enhanced cytosolic delivery, and thus ensured precise mRNA delivery and release.

Our study showed that the DCMNP vaccine elicited humoral and cellular immune responses against infections with superior safety. DCMNPs selectively induced strong mRNA expression in the spleen and lymph nodes. In addition to producing strong IgG antibodies and neutralizing antibodies in immunized mice, DCMNPs also induced CD4^+^ and CD8^+^ T cells. Moreover, DCMNPs efficiently migrated to lymph nodes and generated potent antigen‐specific T cell responses. Overall, our DC‐mimicking nanoparticle delivery system, with high biocompatibility and transfection efficiency, offers a promising prospect for mRNA delivery.

## 4 Experimental Section

### Ethics Statement

All of the animal procedures were reviewed and approved by the Animal Experiment Committee of the Laboratory Animal Center, Academy of Military Medical Sciences, China (Assurance Number: IACUC‐DWZX‐2022‐606).

### Cell Lines and Mice

HEK293T cells were cultured in Dulbecco's Modified Eagle's Medium (DMEM; Gibco) containing 10% fetal bovine serum (FBS) (HyClone; USA) and 100 U mL^−1^ penicillin‐streptomycin solution (HyClone, USA). DC2.4 cells were cultured in RPMI‐1640 (Gibco, USA) containing 10% FBS and 100 U mL^−1^ penicillin‐streptomycin solution. HEK293T‐ACE2 were cultured in DMEM containing 10% FBS, 100 U mL^−1^ penicillin‐streptomycin and a solution of 0.75 µg mL^−1^ Puromycin. Specific pathogen‐free (SPF) female BALB/c mice were purchased from Vital River Laboratory Animal Technology Co., Ltd. (Beijing, China) and were maintained under standard conditions with a 12/12 h light/dark cycle with free access to food and water. All animal studies were performed in strict accordance with the guidelines set by the Chinese Regulations of Laboratory Animals and Laboratory Animal Requirements of Environment and Housing Facilities.

### Synthesis of SARS‐CoV‐2 Omicron Spike protein mRNA‐4138

mRNA‐4138 was designed and synthesized based on the Spike protein of SARS‐CoV‐2. mRNA‐4138 was synthesized using the T7‐FlashScribe^TM^ Transcription kit (CellScript) in vitro. A cap 1 structure was produced using the ScriptCap™ Cap 1 Capping System kit (CellScript) according to the manufacturer's instructions. Then, the mRNA‐4138 was purified by ammonium acetate precipitation and resuspended in RNase‐free water. The quality and concentration of mRNA‐4138 was verified using an Agilent 2100 bioanalyzer and Agilent RNA Nano 6000 assay kit.^[^ ^24^ ^]^

### BMDC Extraction and Pre‐Engineering

BMDCs were extracted from mice as previously described.^[^ ^16^, ^25^ ^]^ BALB/c mice were sacrificed, and the femur was separated from the tibia by cutting the connection point. The muscle and connective tissue were removed from the femurs and tibias. The epiphyses were removed by cutting the tips of the bone with scissors and injecting complete RPMI‐1640 medium to flush out the marrow into a petri dish containing complete RPMI‐1640 medium. The RPMI‐1640 and bone marrow cell solution were passed through a 70 µm cell strainer. The media and cell mixture were centrifuged at 1,500 rpm for 5 min. Then, 10 mL of sterile PBS was added to resuspend the cell pellet. The PBS and cell mixture was centrifuged at 1,500 rpm for 5 min. The cell pellets were resuspended in RPMI‐1640 containing 10% FBS and granulocyte‐macrophage colony‐stimulating factor (GM‐CSF, 20 ng mL^−1^) and cultured at 37 °C and 5% CO_2_. Cells were centrifuged after 72 h and resuspended in 20 mL of complete RPMI‐1640 medium with GM‐CSF (10 ng mL^−1^) and IL‐4 (10 ng mL^−1^). At day 6, cells were treated with R848 (40 ng mL^−1^) for maturation for 48 h. Subsequently, mature DCs were collected and the mature markers of DCs were measured by flow cytometry.

### Isolation of the DC Membrane

According to a previously reported method.^[^ ^26^ ^]^ DCs were collected, and washed three times with cold PBS and centrifuged at 1,000 rpm for 5 min. Cell pellets were suspended in a hypotonic lysis buffer and disrupted by sonication in an ice bath for 15 min, followed by centrifuging at 700 × g for 5 min at 4 °C to obtain the cell membranes in the supernatant. The resultant supernatant was further ultracentrifuged at 50 000 rpm for 30 min at 4 °C to obtain cell membranes, which were then rehydrated in DNase/RNase‐free water. Then the DC membranes were extruded through 450 and 200 nm filters. The protein concentrations of the DC membrane suspensions were determined using bicinchoninic acid (BCA) assays. The presence of DCM was verified through a morphological examination by TEM (Hitachi H‐7650, Tokyo, Japan) using a negative stain technique and the size was measured using a Litesizer 500 (Anton Paar) instrument.

### Preparation and Characterization of DCMNPs

The ionizable lipid (YK009) was synthesized according to previous reports.^[^ ^24b^ ^]^ Then YK009 and 1,2‐dioleoyl‐sn‐glycero‐3‐phosphoethanolamine (DOPE) were dissolved in ethanol at 10 µg µL^−1^. The mass ratio of YK009 and DOPE was 5:6. The mRNA was stocked in DNase/RNase‐free water at 1 µg µL^−1^. The mRNA was dissolved in 250 mM citrate buffer at 0.1 µg µL^−1^. The lipid mixture was combined with mRNA at a mass to volume ratio of 25:11 (mRNA: lipid mixture). Subsequently, the different volumes of DCM were combined with mRNA/YK009/DOPE complexes, introduced into a mini extruder (Avanti Polar Lipids), and sonicated for 15 min (40 kHz) to further homogenize and promote membrane fusion. As reported previously, the fusion between liposomes and cell membranes might be due to the lipid structure of these two materials.^[^ ^27^ ^]^ The optimal formulation was determined with Fluc mRNA by transfection of HEK293T and DC2.4 cells.

To characterize DCMNPs, the DCMNPs size and zeta potential were measured by a Litesizer 500 instrument, and the morphology of DCMNPs was characterized by TEM. The encapsulation efficiency was analyzed with a RiboGreen assay according to the manufacturer's instructions. Briefly, DCMNPs were diluted with TE buffer and treated with 2% Triton X‐100 to release the mRNA at 37 °C for 1 h. Subsequently, untreated DCMNPs, Triton X‐100 treated DCMNPs and a series of 100 µL mRNA standards were transferred to a 96‐well plate and incubated with 100 µL of diluted RiboGreen reagent at 37 °C for 40 min. Following incubation, the fluorescence intensity of the wells was recorded by an I‐control Infinite 200 PRO microplate reader (TECAN). Encapsulation efficiency (%) = [(total mRNA – free mRNA)/total mRNA] × 100%.^[^ ^28^ ^]^ Additionally, a western blot assay was performed to detect the presence of major functional membrane proteins (CD80, CD86, and MHC I) on the DCMNPs.

### Cytotoxicity of DCMNPs In Vitro

To test the potential cytotoxicity of the DCMNP vaccine, HEK293T and DC2.4 cells were seeded on a 96‐well plate at a seeding density of 2 × 10^4^ cells well^−1^, then treated with DCMNPs (0.1 µg mRNA‐4138). The cell viability was assayed 24 h later using a Cell Counting Kit‐8 assay (CCK‐8, Dojindo Laboratories, Kumamoto, Japan).

### Protein Expression of DCMNPs In Vitro

HEK293T and DC2.4 cells were tested for the protein expression of mRNA delivered by the DCMNPs. Cells were seeded on a 12‐well plate at a seeding density of 2 × 10^5^ cells well^−1^. After 24 h, cells were incubated with DCMNPs (1.5 µg eGFP mRNA) for 24 h, and the eGFP expression was visualized using an Olympus IX71 fluorescent microscope (Nikon Corporation, Tokyo, Japan).

SARS‐CoV‐2 Spike protein expression was analyzed by western blotting. Briefly, cells were seeded on a 6‐well plate at a seeding density of 2 × 10^6^ cells well^−1^. After 24 h, cells were incubated with DCMNPs (3 µg mRNA‐4138) for 24 h. The Spike protein was detected by western blotting with a mouse monoclonal antibody against the SARS‐CoV‐2 Spike protein (Sino Biological).

The expression of SARS‐CoV‐2 Spike protein in cells was detected by an indirect immunofluorescence assay. Briefly, cells were seeded on 35 mm glass‐bottom culture dishes and cultured for 24 h. Then cells were incubated with DCMNPs (1.5 µg mRNA‐4138) for 24 h. Subsequently, the cells were washed with cold PBS, and fixed with 4% paraformaldehyde for 30 min, and permeabilized with 0.5% Triton X‐100 for 15 min. Next, the cells were washed and blocked with Immunol staining blocking buffer for 30 min. Then, the cells were washed and incubated with SARS‐CoV‐2 Spike protein primary antibody overnight at 4 °C. Following incubation, the anti‐mouse secondary antibody conjugated with Alexa Fluor 488 (Thermo Fisher) were added and incubated for 1 h. Finally, the cells were incubated with counterstained with 4,6‐diamidino‐2‐ phenylindole (DAPI) for 15 min. The images were captured using a laser confocal microscope (Olympus, Japan).

### Endosomal Escape

We investigated the lysosome escape behaviors of the MFP‐488 fluorescently labeled mRNA‐4138 and the lysosomes stained with Lysotracker red in DC2.4 cells using a high content imaging system (PerkinElmer Opera Phenix). Briefly, DC2.4 cells were seeded on a 96‐well plate at a seeding density of 2 × 10^4^ cells/well and treated with DCMNPs (0.1 µg MFP‐488 fluorescently labeled mRNA‐4138) and incubated for 1, 3, 6, and 12 h, and lysosomes were labeled with Lysotracker red at the corresponding time points, the cell nuclei were stained with Hoechst 33 342. The colocalization/lysosomal area was analyzed by high content imaging system. Furthermore, to investigate the intracellular distribution of mRNA, HEK293T and DC2.4 cells were incubated with DCMNPs (0.1 µg MFP‐488 fluorescently labeled mRNA‐4138) for 6 h, before staining the cell lysosomes with Lysotracker red, and the cell nuclei with Hoechst 33 342. The images were captured using a laser confocal microscope.

### In Vivo Imaging of DCMNPs

To test the capability of DCMNP vaccine delivery and biodistribution in vivo, BALB/c mice were inoculated with DCMNPs (10 µg FLuc mRNA) via intramuscular or intravenous injection, while another group of mice received the same dose of FLuc mRNA encapsulated in MC3‐LNP. Under isoflurane anesthesia, mice were intraperitoneally injected with luciferase substrate at different times post inoculation. Bioluminescence signals were collected at 3, 6, and 24 h after DCMNPs administration by an IVIS Spectrum instrument (PerkinElmer). The bioluminescence signals in the regions of interest (ROIs) were quantified using Living Image. Major organs (heart, liver, spleen, lung, kidney) and lymph nodes were harvested 3 h after injection, and the bioluminescence signals were collected by an IVIS Spectrum instrument.

### Vaccination of DCMNPs in Mice

Female BALB/c mice were immunized intramuscularly with DCMNPs (mRNA‐4138 10 µg, n = 5; 30 µg, n = 5) every 14 days after initial immunization using a three‐dose vaccination strategy. The negative control mice received the same volume of PBS. Sera were collected on days 10, 24, and 38 after initial immunization for detection of SARS‐CoV‐2 Spike‐specific IgG binding antibodies and neutralizing antibodies against SARS‐CoV‐2 pseudovirus infection. Spleen tissues were collected at day 42 after initial immunization to evaluate the cellular immune responses by ELISPOT and flow cytometry.

### Sera Antibody Titer Evaluation

SARS‐CoV‐2 Spike‐specific IgG antibody titers were determined by ELISA. Briefly, 1 µg mL^−1^ recombinant SARS‐CoV‐2 Spike protein was coated onto 96‐well plates overnight at 4 °C. The plates were blocked with 2% albumin for 2 h and washed five times with TBST (0.2% Tween‐20). Mouse immune serum samples were diluted in blocking buffer and incubated for 2 h at 37 °C. After five washes with wash buffer, horseradish peroxidase (HRP)‐conjugated goat anti‐mouse IgG (Beyotime) was added to the plate and incubated for 1 h at 37 °C. After washing the plates five times, chromogen solution was added and incubated for 20 min at 37 °C. The endpoint titers were defined according to the manufacturer's instructions.

### Pseudovirus Neutralization Assay

The SARS‐CoV‐2 pseudovirus neutralization assay was performed as previously reported.^[^ ^29^ ^]^ Briefly, three‐fold serially diluted mouse immune serum samples were mixed with SARS‐CoV‐2 pseudovirus (omicron BA.1) and incubated at 37 °C for 1 h. Next, the mixture and HEK293T‐ACE2 cells were seeded on 96‐well plates (20000 cells/well) and incubated at 37 °C and 5% CO_2_ for 24 h. Subsequently, 100 µL of luciferase substrate was added to each well and incubated at room temperature protected from light for 5 min. Finally, luciferase activity was measured; the 50% neutralization titer (NT_50_) was defined as the serum dilution of the relative light units (RLUs) reduced by 50% compared to the virus control wells.

### ELISPOT Assay

The cellular immune responses in the vaccinated mice were assessed using IL‐2, IL‐4, IL‐6 and IFN‐γ precoated ELISPOT kits (MabTech), according to the manufacturer's protocol. Briefly, the ELISPOT plates were washed four times with PBS, blocked with RPMI‐1640 containing 10% FBS, and incubated for at least 1 h at room temperature. The medium was removed and the immunized mice splenocytes were plated at 5 × 10^5^ cells well, stimulated with a SARS‐CoV‐2 peptide pool (5 µg mL^−1^ of each peptide; Miltenyi Biotec), and incubated at 37 °C and 5% CO_2_ for 48 h. Following incubation, the cells were removed, and the plates were washed five times with PBS. Biotinylated IL‐2, IL‐4, IL‐6, or IFN‐γ detection antibody was added (100 µL well^−1^), incubated for 2 h at room temperature. The plates were washed five times with PBS, before adding streptavidin‐HRP was added (100 µL well^−1^) and incubating for 1 h at room temperature. Subsequently, TMB substrate solution was added to allow distinct spots to emerge, and then rinsed with deionized water to stop color development. The spots were inspected and counted using an automated VSR07 ELISPOT reader (AID).

### Flow Cytometry Analyses for Mouse Splenocytes

Memory T cell proliferation in immunized mice was evaluated using a FACS Aria II flow cytometer.^[^ ^30^ ^]^ Briefly, a total of 5 × 10^5^ immunized mice splenocytes were seeded onto 96‐well plates, stimulated with a SARS‐CoV‐2 peptide pool, and incubated at 37 °C and 5% CO_2_ for 12 h. Brefeldin A (5 µg mL^−1^) was added to the mouse splenocytes and incubated for 4 h. Then, CD16/CD32 antibodies were used to block the Fc receptors of cells for 15 min. The splenocytes were stained with fluorescently conjugated antibodies to CD3 (PE/Cyanine7), CD4 (FITC), CD8 (PercP), CD44 (PE), and CD62L (APC), and incubated for 30 min at 4 °C. The cells were washed with cell staining buffer and the dead cells were stained with Fixable Viability Dye eFluor™ 780 (Thermo Fisher) for 30 min at 4 °C. Finally, the cells were washed with cell staining buffer, and the data were collected by a FACS Aria II flow cytometer. Tfh and GC B cells in the lymph nodes were detected by flow cytometry.^[^ ^18^, ^31^ ^]^

### In Vivo Toxicity

To evaluate the toxicity of the DCMNP vaccine in vivo, the level of several important cytokines and the liver and kidney function were analyzed 24 h post vaccination in mice vaccinated with DCMNPs (30 µg mRNA‐4138). For histopathology, organ tissues, including the heart, liver, spleen, lung, and kidney, were extracted at 24 h post‐inoculation, before staining with H&E for histopathological examination.

### Statistical Analysis

Statistical analyses were performed using GraphPad Prism 8.0 (GraphPad Software). All of the data are presented as the mean ± SEM. Statistical differences were analyzed by one or two‐way ANOVA with multiple comparison tests. All tests were accepted as statistically significant when *p* < 0.05.

## Conflict of Interest

The authors declare no conflict of interest.

## Supporting information

Supporting Information

## Data Availability

The data that support the findings of this study are available from the corresponding author upon reasonable request.
