# Supplementary material for: Dendritic Cell‐Mimicking Nanoparticles Promote mRNA Delivery to Lymphoid Organs
Source: Adv Sci (Weinh). 2023 Oct 22;10(33):2302423. doi: 10.1002/advs.202302423 (PMC10667832; doi:10.1002/advs.202302423)
Supplement: Supplementary file 1 — Supporting Information [file ADVS-10-2302423-s001.pdf]

ADVANCED SCIENCE
Open Access

Supporting Information

for *Adv. Sci.*, DOI 10.1002/advs.202302423

Dendritic Cell-Mimicking Nanoparticles Promote mRNA Delivery to Lymphoid Organs

Yiming Cao, Jinrong Long, Huisheng Sun, Yiqi Miao, Ye Sang, Haitao Lu, Changxiao Yu, Zhen Zhang, Lin Wang, Jing Yang* and Shengqi Wang**

Supporting Information

Dendritic cell-mimicking nanoparticles promote mRNA delivery to lymphoid organs

Yiming Cao[1,2#]; Jinrong Long[1#]; Huisheng Sun[1#]; Yiqi Miao[1#]; Ye Sang[1]; Haitao Lu[1]; Changxiao Yu[1]; Zhen Zhang[1]; Lin Wang[2]*; Jing Yang[1]*; Shengqi Wang[1]*

1. Y. Cao, J. Long, H. Sun, Y. Miao, Y. Sang, H. Lu, C. Yu, Z. Zhang, J. Yang, S. Wang

Bioinformatics center of AMMS, Beijing 100850, P.R.China.

E-mail: sqwang@bmi.ac.cn; jingyang0511@sina.com;

2. Y. Cao, L. Wang

Beijing Institute of Radiation Medicine, Beijing 100850, P.R.China.

E-mail: wanglin07@sina.com

[#] Y. Cao, J. Long, H. Sun, and Y. Miao contributed equally to this work.

Supplementary Experimental Section

Materials: Dlin-MC3-DMA was purchased from AVT (Shanghai) pharmaceutical tech Co., Ltd. Cholesterol and 1,2-distearoyl-sn-glycero-3-phosphocholine (DSPC) were purchased from SIGMA. 1,2-dimyristoyl-rac-glycero-3-methoxypolyethylene glycol-2000 (DMG-PEG2000) was purchased from Avanti Polar Lipids. 1,2-dioleoyl-sn-glycero-3-phosphoethanolamine (DOPE) and resiquimod (R848) were purchased from TOKYO chemical industry Co., Ltd. Firefly luciferase mRNA (Fluc mRNA) and enhanced green fluorescent protein mRNA (eGFP mRNA) were purchased from Trilink Biotechnologies.

Flow cytometric analysis of surface markers

At the immature stages of development, DCs act as sentinel cells. After DCs matures, upregulation of antigen processing and presentation, and expression of costimulatory molecules (high CD80 and CD86).[1] We detected the expression of CD80 and CD86 by flow cytometry. All groups of DC (Immature DC alone, Immature DC stimulated with 40 ng·mL^{-1} R848 for 48 h) surface marker expression was assessed using a FACS Aria II flow cytometer. Briefly, 5×10^5 cells were aliquoted into tubes and washed once washed with cell staining buffer. Then, CD16/CD32 antibodies were used to block the Fc receptors of cells for 15 min. Cells were stained with fluorescently conjugated antibodies to CD80 (PE) and CD86 (APC), and incubated for 30 min at 4°C. Finally, the cells were washed with cell staining buffer, and data were collected by a FACS Aria II flow cytometer.

Evaluation of eGFP expression

HEK293T and DC2.4 cells were tested for protein expression of mRNA delivered by the DCMNPs. Cells were seeded on a 12-well plate at a seeding density of 2×10^5 cells/well. After 24 h, cells were incubated with DCMNPs (1.5 μg eGFP mRNA) for 24 h. The collected cells were analyzed with a flow cytometer. The data analysis was performed by a FACS Aria II flow cytometer.[2]

Evaluation of Fluc expression

HEK293T and DC2.4 cells were seeded on a 96-well plate at a seeding density of 2×10^4 cells/well and treated with DCMNPs (0.1 μg Fluc mRNA) for 24 h. After incubation, 100 μL luciferase substrate (PerkinElmer) was added to each well, followed by incubation for 5 min in darkness. The luciferase signals were detected by an I-control Infinity 200 PRO microplate reader (TECAN).

References

[1] A. Mihret, G. Mamo, M. Tafesse, A. Hailu, S. Parida, *BMC research notes* **2011**, 4, 247.
[2] T. Nakamura, T. Nakade, Y. Sato, H. Harashima, *International Journal of Pharmaceutics* **2023**, 636, 122810.

Supplementary Figures

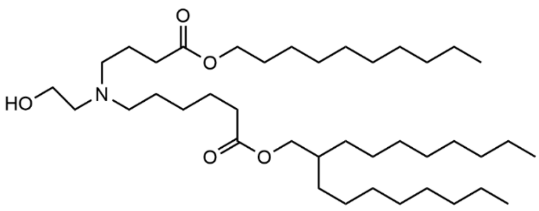

Figure S1. The structure of the novel ionizable lipid (YK009).

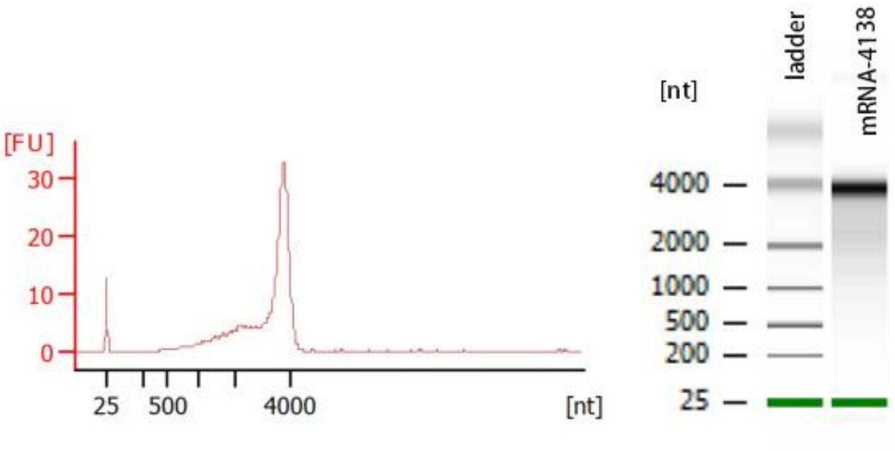

Figure S2. The electropherogram of SARS-CoV-2 Omicron Spike protein mRNA-4138. Bioanalysis of SARS-CoV-2 Omicron Spike protein mRNA-4138 was performed using the Agilent 2100 bioanalyzer system.

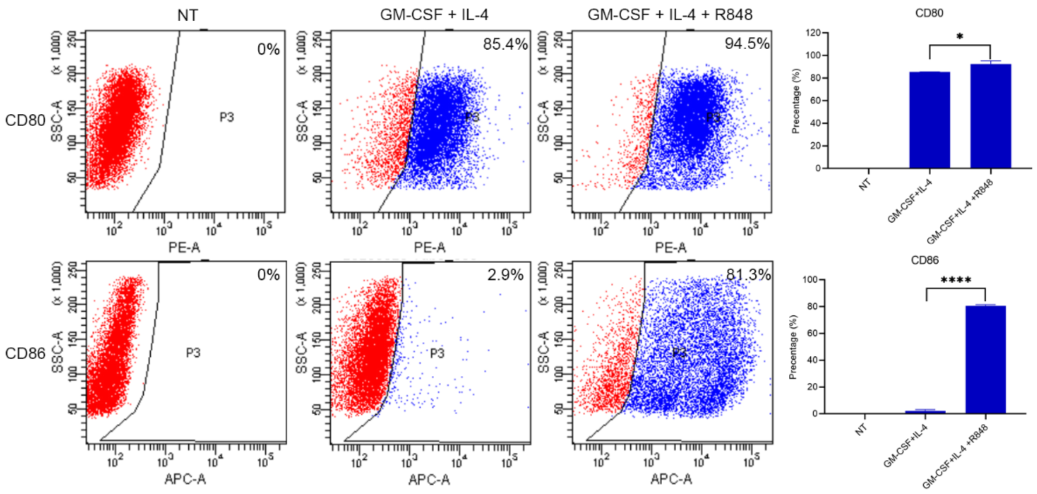

Figure S3. The expression of DCs maturation markers was detected by flow cytometry using CD80 (PE) and CD86 (APC). Data are shown as the mean ± SEM (n = 3) and were analyzed by one-way ANOVA with multiple comparisons tests (*$p < 0.05$; ****$p < 0.0001$).

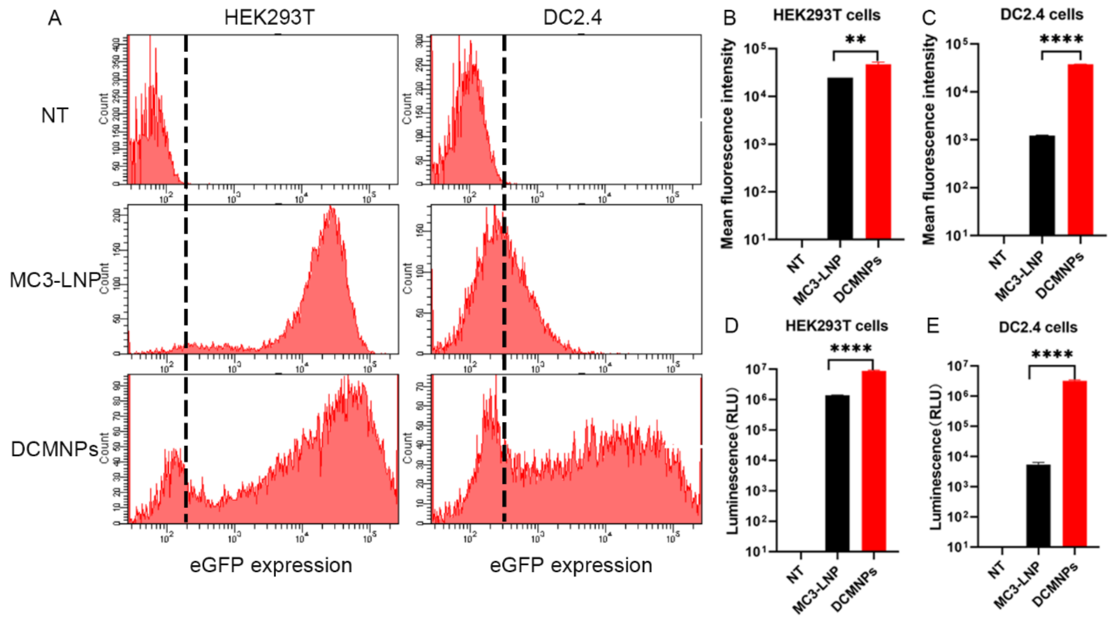

Figure S4. mRNA expression intensity and introduction efficiency at each cell level. (A) Typical histograms of eGFP expression by a FACS Aria II flow cytometer analysis. (B) Mean fluorescence intensity of eGFP in HEK293T cells. (C) Mean fluorescence intensity of eGFP in DC2.4 cells. (D) Fluc expression in HEK293T cells. (D) Fluc expression in DC2.4 cells. Data are shown as the mean ± SEM (n = 3) and were analyzed by one-way ANOVA with multiple comparisons tests (**$p < 0.01$; ****$p < 0.0001$).

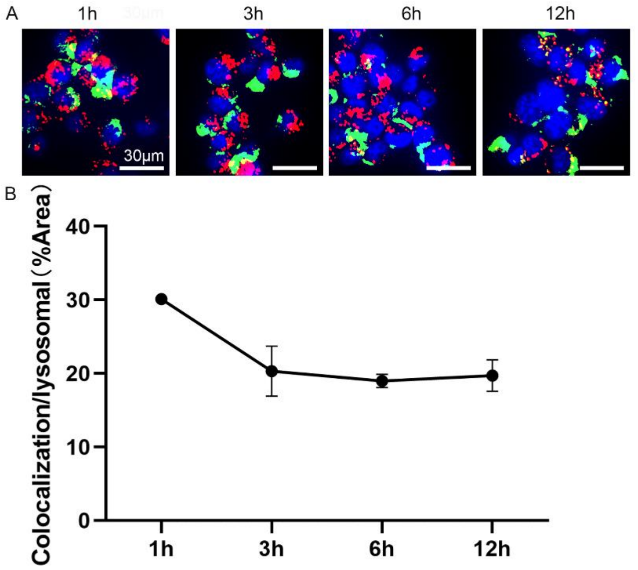

Figure S5. Lysosomal escape. (A) The nanoparticles were incubated with DC2.4 cells for 1, 3, 6, and 12 h, respectively. The lysosomes were labeled with Lysotracker red

(red), the mRNA-4138 were labeled with MFP-488 fluorescently (green) and the cell nuclei were labeled with Hoechst 33342 (blue). Scale bar: 30 μm. (B) The Colocalization/lysosomal area values were obtained and analyzed by high content imaging system.

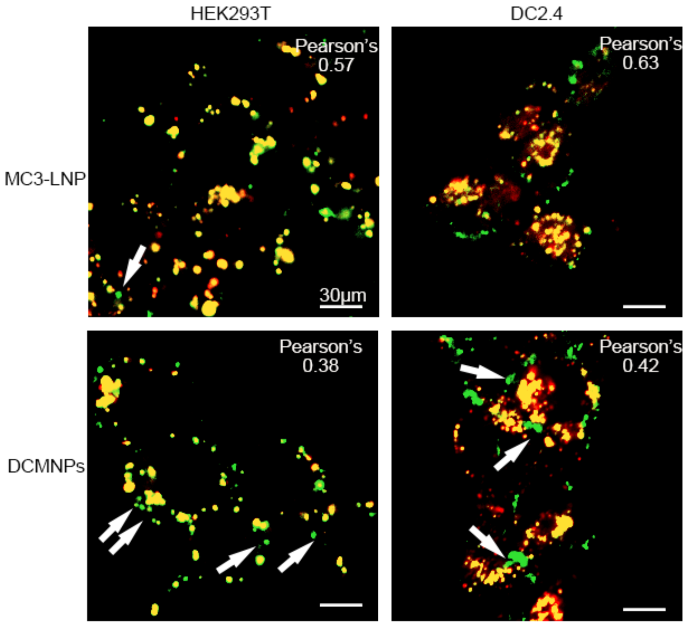

Figure S6. Confocal images of showing intracellular distribution of mRNA (green) and lysosomes (red) at 6 h post transfection in HEK293T and DC2.4 cells. The white arrow indicates the lysosomal escape of the delivered mRNA. Scale bar: 30 μm.
